# Using deep maxout neural networks to improve the accuracy of function prediction from protein interaction networks

**DOI:** 10.1371/journal.pone.0209958

**Published:** 2019-07-23

**Authors:** Cen Wan, Domenico Cozzetto, Rui Fa, David T. Jones

**Affiliations:** 1 Bioinformatics Group, Department of Computer Science, University College London, London, United Kingdom; 2 Biomedical Data Science Laboratory, The Francis Crick Institute, London, United Kingdom; Cincinnati Children’s Hospital Medical Center, UNITED STATES

## Abstract

Protein-protein interaction network data provides valuable information that infers direct links between genes and their biological roles. This information brings a fundamental hypothesis for protein function prediction that interacting proteins tend to have similar functions. With the help of recently-developed network embedding feature generation methods and deep maxout neural networks, it is possible to extract functional representations that encode direct links between protein-protein interactions information and protein function. Our novel method, STRING2GO, successfully adopts deep maxout neural networks to learn functional representations simultaneously encoding both protein-protein interactions and functional predictive information. The experimental results show that STRING2GO outperforms other protein-protein interaction network-based prediction methods and one benchmark method adopted in a recent large scale protein function prediction competition.

## Introduction

The realisation of the complex relationships between genotypes and phenotypes has been fostering the collection and analysis of genome-wide datasets of molecular interactions detected from patterns of physical binding, transcript co-expression, mutant phenotypes, etc. Many specialised databases exist to store and integrate such heterogeneous data at different levels of biological complexity. At one end of the scale, the International Molecular Exchange (IMEx) consortium gathers non-redundant protein-protein interactions (PPIs) from peer-reviewed scientific publications, and provides manually curated details about the experimental conditions [1]. At the opposite end, several resources extend these primary data with indirect or predicted associations to paint a more complete picture for whole organisms [2–5]. For instance, STRING [5] considers experimentally detected PPIs, conserved mRNA co-expression, co-mention in abstracts and papers, interactions from curated databases, conserved gene proximity, gene co-occurrence/co-absence and gene fusion events. Interactions in such databases are typically assigned confidence scores, which can be used for integration purposes [2, 6, 7]. Not only these data provide valuable direct links between genes and their biological roles, but also form the basis for protein function prediction methods that do not rely on traditional annotation transfers from sequence. Omics data have long offered a suitable opportunity by lending themselves to network representations, where genes or protein products are nodes and edges represent molecular interactions. This modelling approach can be easily exploited using the “guilt-by-association” principle: if the edges reflect biological facts reliably, adjacent nodes have more similar functions than those further away in the network—e.g. because they form a macromolecular complex, or their activities are coordinated in a specific biological process.

The earliest methods therefore transfer annotations from nodes that are either adjacent or within close distance, possibly taking into account the enrichment of the functional labels [8]. Because the network topology is far from uniform and different functions arise from unevenly sized gene sets, using one particular distance or number of neighbours inevitably affects prediction accuracy. More sophisticated algorithms therefore try to group the nodes into functional modules or communities—each associated with a given function—and then make annotation transfers within them [9–14]. The preliminary identification of functionally coherent subgraphs, however, poses additional challenges, which can make module-assisted predictors less accurate than those based on neighbour counting [15]. Alternatively, the functional annotations can be transferred via PPI parters’ homologous proteins. For example, Zhang et al. (2018) [16] proposed a method, namely PPI-homolog, to transfer the functional annotations of multiple homologs of the target protein’s interaction partners to make the function prediction. More recently, network propagation methods have become increasingly popular to address a wide range of problems [17]. They broadcast annotations from labelled proteins to others by running random walks, which visit the nodes in the network randomly until stopping criteria are met [18–20]. If the edges are weighted, this information controls the probability of traversing them; otherwise equal probabilities are used. Because the propagation is affected by node degree and edge weights, this approach reduces the chance of erroneous predictions from highly multifunctional hub proteins to adjacent nodes, which perform fewer functions. Alternatively, the transition probabilities can be used to encode directly the nodes as multi-dimensional features, and thus to make functional annotations with nearest neighbour strategies [21, 22]. Cho et al. (2016) [23] and Gligorijević et al. (2018) [24] have instead used them to embed the STRING networks jointly—that is to map nodes to continuous features, which best explain the transition probabilities and the graph topology. The usefulness of the resulting features has been demonstrated for the task of protein function prediction.

This study proposed a novel PPI network-based protein function predicting method, STRING2GO. It adopts deep maxout neural networks to learn a novel type of functional biological network feature representations simultaneously encapsulating both node neighborhoods and co-occurrence functions information. These higher-level representations are learnt in a supervised way by training deep maxout neural networks to output all the terms in biological process domain associated with an input protein—an approach that has led to higher predictive accuracy in the past [25, 26]. The experimental results show that STRING2GO significantly outperforms other PPI network embedding-based protein function prediction methods.

## Materials and methods

### Data collection

Firstly, human proteins were retrieved from the UniProtKB/SwissProt release 2017_05 [27], while the corresponding protein-protein interactions information was retrieved from STRING v10.0 [28] that includes seven component networks from heterogeneous data sources and one integrated network. The mapping between UniProtKB/SwissProt accession numbers and Ensembl protein identifiers adopted in STRING was obtained by using the Biomart tool [29].

Experimentally supported Gene Ontology (GO) term annotations—identified with evidence code EXP, IDA, IPI, IMP, IGI or IEP—were collated from the UniProtKB/SwissProt release 2017_05 and UniProt-GOA release 168 [30], and propagated over “is a” relationships in the Gene Ontology database [31]—GO OBO file release 2017-04-28. To assure the feasibility of the following machine-learning experiments, only biological process (BP) annotating at least 100 proteins were initially considered. To guarantee that the predictions are sufficiently specific and informative, this list was subsequently filtered so that only the deepest terms in the ontology were retained—i.e. the terms a and b were kept if and only if there are no “is_a” paths from a to b and from b to a. These steps yielded a vocabulary consisting of 204 BP terms (detailed information is included in S1 Table).

The set of human proteins was split into a large subset for GO term-specific classifier training and a small subset for hold-out evaluation. 10,667 proteins with at least one cellular component term were initially selected from the whole set. Out of these, 1,000 proteins were randomly selected for hold-out evaluation from the subset of well-annotated entries—i.e. those with at least 28, 5 and 14 experimental or electronic biological process, molecular function and cellular component terms respectively. After removing electronic annotations, the hold-out set for BP terms contains 982 proteins, while the large set contains 5,000 proteins. We also create a separated protein-set for a temporal validation by selecting 428 proteins who had no experimental annotation by any 204 BP terms but received at least one after 6 months. The source files were collected from UniProtKB/SwissProt release 2017_11, UniProt-GOA release 174 and GO OBO file 2017-10-30. In order to further evaluate the performance of our methods on predicting homology-independent proteins, we further removed the homologous proteins in the hold-out and temporal validation protein-sets respectively by using BLAST searches against the training protein-set with different E-value thresholds, i.e. 10^−5^, 10^−4^, 10^−3^ and 10^−2^, leading to different degrees of homolog-removal subsets of the original hold-out and temporal validation protein-sets. The higher value of the E-value threshold denotes the wider definition of protein homology, leading to a more stringent condition on the evaluation of the homology-independent prediction. The hold-out protein-sets with different E-value thresholds range from 255 to 192 proteins, while the temporal validation protein-sets with different E-value thresholds range from 198 to 182 proteins. The detailed information is included in the S2 Table.

### Predictive performance evaluation

Predictive performance was evaluated on the ability to annotate both individual labels (GO term-centric) and protein function (protein-centric), following the methodology adopted in [32]. For the GO term-centric evaluation, we calculate the F_1_, Matthews Correlation Coefficient (MCC), and Area Under Precision Recall Curve (AUPRC) scores for evaluating the predictive performance of the GO term-specific classifier on the hold-out protein-set. In details, the GO term-centric F_1_ (i.e. F_1_GO_) score is used for evaluating the performance of methods when predicting protein annotations for individual GO terms. As shown in Eq 1, the F_1_ score is obtained by calculating the harmonic mean of precision and recall values. The precision value (Eq 2) is calculated by dividing the number of true positive (TP) predictions over the summation of true positive and false positive (FP) predictions, while the recall value (Eq 3) is calculated by dividing the number of true positive (TP) predictions over the summation of true positive and false negative (FN) predictions. The MCC score that is calculated by Eq 4 is widely used for evaluating the performance of prediction methods on data, where the proportion of binary class labels is highly imbalanced. Analogously, the AUPRC score is also a well-known metric for evaluating the performance of prediction methods on imbalanced class prediction tasks.

$$\begin{array}{c} F_{1}=2\times \frac{\text{Precision}\times \text{Recall}}{\text{Precision}+\text{Recall}} \end{array}$$ (1)

$$\begin{array}{c} \text{Precision}=\frac{\text{TP}}{\text{TP}+\text{FP}} \end{array}$$ (2)

$$\begin{array}{c} \text{Recall}=\frac{\text{TP}}{\text{TP}+\text{FN}} \end{array}$$ (3)

$$\begin{array}{c} \text{MCC}=\frac{\text{TP}\times \text{TN}-\text{FP}\times \text{FN}}{\sqrt{\left(\text{TP}+\text{FP})(\text{TP}+\text{FN})(\text{TN}+\text{FP})(\text{TN}+\text{FN}\right)}} \end{array}$$ (4)

For the protein-centric evaluation, we calculate the F_max_ score by predicting the GO term annotations for the hold-out and different degrees of homolog-removal hold-out evaluation using the trained GO term-specific classifiers. The F_max_ score is used by CAFA experiments [32] for evaluating the performance of methods when predicting GO term annotations for all protein samples. As shown in Eq 5, the F_max_ score is obtained by choosing the maximum averaged F_1_ score over all protein samples’ GO term annotation prediction, according to the varied decision threshold. The averaged F_1_ score for threshold *τ* is calculated by the averaged precision $\bar{{\text{Precision}}_{\tau }}$ (Eq 6) and recall $\bar{{\text{Recall}}_{\tau }}$ (Eq 7) values. The $\bar{{\text{Precision}}_{\tau }}$ value is calculated by the total amount of precision values for the GO term annotation predictions of all protein sequences *S*, over the number of protein sequence *m* with at least one GO term annotation predictive posterior probability being equal or greater than the value of threshold *τ*. Analogously, the $\bar{{\text{Recall}}_{\tau }}$ value is calculated by the total amount of recall values for the GO term annotation predictions of all protein sequences *S*, over the total number of protein sequences *n*. Then the corresponding *τ* to F_max_ score is used as the prior knowledge to calculate the other type of protein-centric averaged F_1_ score, i.e. F_*τ*_, for the temporal and different degrees of homolog-removal temporal validation. Note that we mainly discuss the F_max_ and F_*τ*_ scores obtained by the homolog-removal protein-sets generated by applying the E-value threshold of 10^−2^, whereas the results for all other different degrees of homolog-removal protein-sets are also reported in the S3 and S4 Tables.

$$\begin{array}{c} F_{\text{max}}={\text{max}}_{\tau }\left\{2\times \frac{\bar{{\text{Precision}}_{\tau }}\times \bar{{\text{Recall}}_{\tau }}}{\bar{{\text{Precision}}_{\tau }}+\bar{{\text{Recall}}_{\tau }}}\right\} \end{array}$$ (5)

$$\begin{array}{c} \bar{{\text{Precision}}_{\tau }}=\frac{1}{m}\sum_{s}\frac{{\text{TP}}_{s,\tau }}{{\text{TP}}_{s,\tau }+{\text{FP}}_{s,\tau }} \end{array}$$ (6)

$$\begin{array}{c} \bar{{\text{Recall}}_{\tau }}=\frac{1}{n}\sum_{s}\frac{{\text{TP}}_{s,\tau }}{{\text{TP}}_{s,\tau }+{\text{FN}}_{s,\tau }} \end{array}$$ (7)

### STRING2GO—A novel protein function prediction method based on learning representations simultaneously encoding the protein-protein interaction and functional annotation information

**Fig 1:**
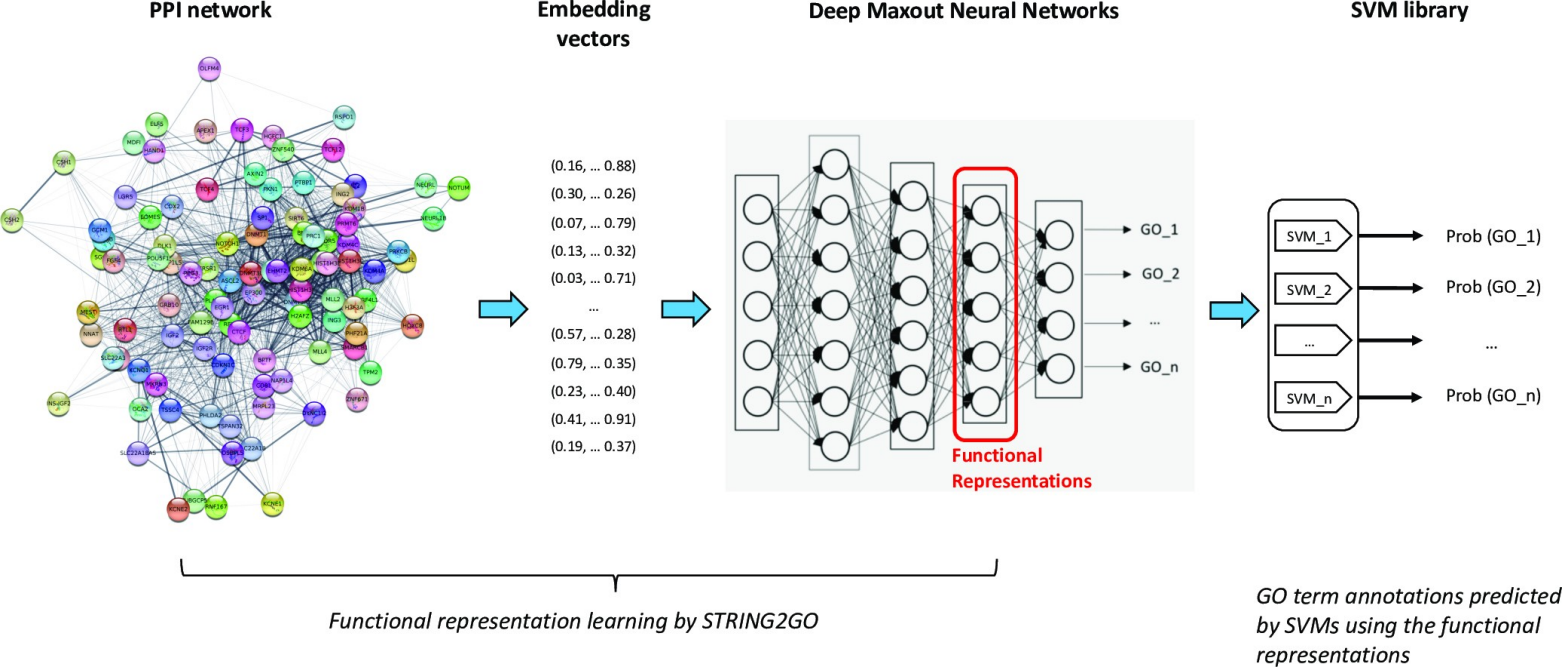
Flow-chart of STRING2GO-based protein function prediction method.

In general, the STRING2GO method is composed of a three-stage machine learning procedure. As shown in the flow-chart of Fig 1, at the first stage, it adopts the network embedding representation generation methods (e.g. Mashup and Node2vec discussed in this work) to generate the vector representations for individual proteins based on the protein-protein interaction network. Then the Deep Maxout Neural Networks (DMNNs) feed-forward those generated representations as the inputs to a set of GO term annotations of individual proteins as the outputs. The new type of functional representations (denoted as STRING2GO_Embedding_) that simultaneously encode the PPI and protein functional annotation information are extracted from the outputs of the 3^*rd*^ hidden layer of DMNNs after finishing the backward propagation optimisation. Finally, STRING2GO trains a library of Support Vector Machines (SVMs) to predict the posterior probability of annotating individual GO terms to the target proteins. Here, we denote this type of STRING2GO method as STRING2GO_Embedding+SVM_ for clarity. In addition, due to the natural functionality of DMNNs, we also propose another type of STRING2GO method, denoted as STRING2GO_Embedding+Sigmoid_, which directly adopts the sigmoid function in the last layer of DMNNs to make predictions.

In this work, we evaluate the predictive performance of our two types of STRING2GO method on predicting the BP terms located in the deep positions in the GO-DAG, benchmarking with the conventional raw network embedding representations-based method, i.e. Embedding+SVM, that merely adopts the raw network embedding representations to train the SVMs for making predictions.

### Network embedding representation generation

In this work, we adopt two types of network embedding representation generation methods, i.e. Mashup [23] and Node2vec [33], to derive representations from STRING networks. Mashup firstly evaluates the diffusion states of nodes in the network by random walks with a restart approach. Then the truncated singular value decomposition is applied to the diffusion state matrix in order to learn a lower dimensional representation space that optimally approximates the original diffusion states information. The usefulness of the resulting network embedding representations has been demonstrated for a range of functional classification tasks, including function and genetic interaction prediction. As suggested, the best-performing Mashup-derived representations are 800 dimensional and generated by the random-walk sampling strategy with the restart probability of 0.5.

Analogously, Node2vec firstly obtains the node neighborhood information by truncated random walks. Then a Skip-gram [34, 35] shallow neural network is used to generate a representation space, where the nodes contain the maximum likelihood of preserving corresponding node neighborhood information. In this work, the neighborhood information was sampled through random walks of length ten, which were biased towards close neighbors by setting the parameter *q* to 2. We also evaluate the performance of representations in different dimensions, i.e. 32, 64, 128, 256 and 512, generated from all different STRING networks [21, 22].

### Deep maxout neural networks training

Deep Maxout Neural Networks (DMNNs) are used for learning the more abstract representations simultaneously encoding the PPI network information and the patterns of term co-occurrence in the biological process functional domain. The network architecture was implemented using the Keras package with Theano backend and consisted of three fully connected hidden layers, followed by an output layer with as many neurons as the numbers of terms selected for the biological process functional domain. Each hidden layer had batch-normalized inputs [36], which were combined through maxout units [37], and were subject to dropout [38] in the course of training. A sigmoid function was used to activate the output neurons.

To limit the computational requirements for model optimization, the initial 10-fold cross validation (with random split of instances) experiments were run in order to identify the best combination of optimizer (AdaGrad), number of maxout units (3), learning rate (0.05), batch size (100 elements), and number of epochs (150), keeping fixed the weight initialisation (Glorot uniform method) and the number of units in all hidden layers, by considering the highest F_1_GO_ scores for predicting all 204 BP terms. Subsequent training stages were aimed at selecting the optimal dimensions of hidden layers that lead to the highest median F_1_GO_ scores (here rounded to two decimal places), from a limited set of options (300, 500, 700 and 1,000). In addition, we also evaluate the predictive performance when using the same dimensions for both input features and the 3^*rd*^ hidden layer outputs. Note that, due to the well-known curse of dimensionality issue [39], if more than two different dimensions of the 3^*rd*^ hidden layer outputs obtain the same median F_1_GO_ scores, we only choose the lowest ones as the optimal dimensions.

### Support vector machine training

Scikit-learn [40] was used to train a set of GO term-specific Support Vector Machines (SVMs) with a radial basis function (RBF) kernel, the parameters of which were identified through a grid search as those maximising the F_1_GO_ score across the stratified 10-fold cross validation experiments. To train each classifier, the set of positive instances consisted of the proteins annotated with the target GO term *t* or its descendants, while the set of negative instances are all remaining proteins not annotated with the target GO term or its descendants. Finally, the well-known Platt scaling method [41] was used to transform the predictive scores of individual SVMs into a probability distribution of binary classes. The data and code can be accessed via https://github.com/psipred/STRING2GO.

## Results

We firstly report the experimental results about evaluating the predictive information included in different STRING networks that are used for generating the raw network embedding representations by two different methods, i.e. Mashup and Node2vec. Then we evaluate the predictive performance of the STRING2GO-learnt functional representation (i.e. STRING2GO_Mashup_ and STRING2GO_Node2vec_) by comparing with their corresponding raw network embedding representations. We also compare the performance of Mashup and Node2vec methods when they are used to generate the raw network embedding representations or be the component methods of STRING2GO to learn the functional representations. Finally, we further compare all prediction methods involved in this work, also comparing with the PPI-homolog [16] and Naïve methods [32].

### Predictive power included in different STRING networks

To begin with, we compare the predictive power of different STRING networks by adopting the Mashup or Node2vec-generated network embedding representations as the inputs of DMNNs for predicting protein function (i.e. STRING2GO_Mashup+Sigmoid_ and STRING2GO_Node2vec+Sigmoid_). Overall, the Combinedscore network-derived embedding representations show the best predictive performance among all different STRING networks-derived ones when using either Mashup or Node2vec methods, while the Textmining network-derived representations also obtain the competitive predictive accuracy. As shown in the 4^*th*^ and 7^*th*^ columns of Table 1, the Combinedscore network-derived representations obtain the highest median F_1_GO_ (hereafter, denoted by ${\tilde{F}}_{1\_\text{GO}}$) scores (0.23 and 0.17) using Mashup and Node2vec respectively. The Combinedscore network also contains the largest number of proteins, interactions and the highest coverage (as shown in the columns 8-10 of Table 1), when mapping the STRING network-included proteins to the training protein-set. The Textmining network-derived representations obtain the second highest ${\tilde{F}}_{1\_\text{GO}}$ score (0.22) using the Mashup method, while also obtain the same highest ${\tilde{F}}_{1\_\text{GO}}$ score (0.17) using the Node2vec method. Moreover, in terms of the predictive information included in other component networks, the Experimental network-derived embedding representations show the third highest predictive accuracy, since they obtain sequentially higher ${\tilde{F}}_{1\_\text{GO}}$ scores than the ones derived by the Database and Coexpression networks respectively. Note that, the embedding representations derived from Neighbourhood, Cooccurrence and Fusion networks show poor predictive performance, since their ${\tilde{F}}_{1\_\text{GO}}$ scores are all equal to zero, and the mapping coverages are all lower than 21.0%. Hereafter, we consider learning the functional representations by STRING2GO only from those 5 networks including relatively rich PPI information and high coverage.

**Table 1:** The optimal dimensions of raw network embedding representations and the corresponding 3^*rd*^ hidden layer outputs (a.k.a. the STRING2GO-learnt functional representations) with their corresponding predictive power for biological process terms prediction, and the main characteristics of different STRING networks.

STRING Networks	Mashup			Node2vec			No. Proteins	No. Interactions	Coverage on Training set
	Input	3^*rd*^ Hidden	${\tilde{F}}_{1\_\text{GO}}$	Input	3^*rd*^ Hidden	${\tilde{F}}_{1\_\text{GO}}$			
Combinedscore	800	800	**0.23**	128	500	**0.17**	**19247**	**8548002**	**93.4%**
Textmining	800	700	0.22	128	1000	**0.17**	19088	7632934	93.3%
Experimental	800	700	0.19	128	1000	0.13	16858	3473862	90.4%
Coexpression	800	700	0.14	256	700	0.09	12774	1537924	72.0%
Database	800	700	0.11	128	700	0.04	7937	424860	56.9%
Neighborhood*	800	300	0.00	32	32	0.00	3514	152248	20.9%
Cooccurrence*	800	300	0.00	32	32	0.00	2754	47478	16.6%
Fusion*	800	300	0.00	32	32	0.00	1495	4120	9.7%

*: Note that those STRING networks obtain 0.00 of ${\tilde{F}}_{1\_\text{GO}}$ scores with all different dimensions, only the lowest dimensions are reported.

We then report the optimal dimensions of network embedding representations derived by Mashup and Node2vec methods from those 5 STRING networks. According to the suggestion in [23], we define 800 as the optimal dimensions for the input network embedding representations derived by Mashup. In terms of the Node2vec-derived network embedding representations, as shown in the 5^*th*^ column of Table 1, 128 are the overall optimal dimensions, since 4 out of 5 network-derived embedding representations in 128 dimensions obtain the highest ${\tilde{F}}_{1\_\text{GO}}$ scores for predicting 204 biological process terms. We then report the optimal dimensions of the STRING2GO-learnt functional representations (a.k.a. the 3^*rd*^ hidden layer outputs of DMNNs) w.r.t. the corresponding optimal dimensions of raw network embedding representation inputs. Generally, STRING2GO encodes the functional predictive information in a high dimensional representation space (ranging from 500–1000 dimensions), when using either Mashup or Node2vec as the raw network embedding representation generation method. As shown in the 3^*rd*^ and 6^*th*^ columns of Table 1, the optimal dimensions of the 3^*rd*^ hidden layer outputs vary between 500 to 1000. Recall that we also evaluate the cases when the dimensions of the 3^*rd*^ hidden layer outputs are the same to the dimensions of raw network embedding representation inputs. None of the functional representations based on Node2vec-derived network embedding representations obtain higher ${\tilde{F}}_{1\_\text{GO}}$ scores when using the same dimensions of inputs as the dimensions of 3^*rd*^ hidden layer outputs, e.g. using 128 as the dimensions of both representation inputs and the 3^*rd*^ hidden layer outputs.

### The functional representations learnt by STRING2GO encode higher predictive power than the corresponding raw network embedding representations

**Fig 2:**
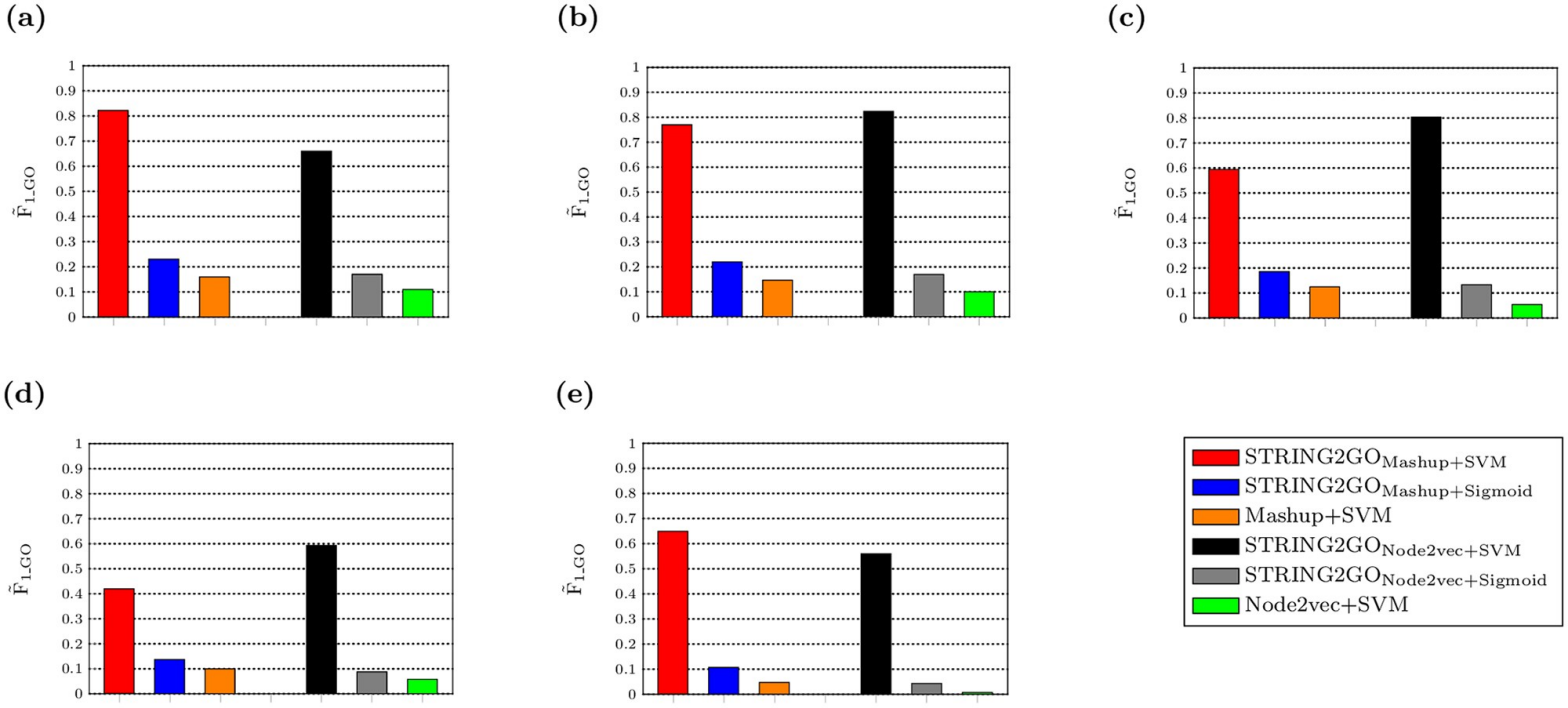
${\tilde{F}}_{1\_\text{GO}}$ scores obtained by network embedding representations and the corresponding STRING2GO-learnt functional representations based on (a) Combinedscore, (b) Textmining, (c) Experimental, (d) Database and (e) Coexpression networks by using SVM or Sigmoid function over the 10-fold cross validation during the GO term-specific classifiers training stage.

We evaluate the predictive performance of STRING2GO-learnt functional representations by conducting pairwise comparisons with the corresponding raw network embedding representations respectively. Generally, in terms of GO term and protein-centric metrics, both STRING2GO_Mashup_ and STRING2GO_Node2vec_ functional representations obtain higher predictive accuracy than Mashup and Node2vec-derived raw network embedding representations. In detail, during the GO term-specific classifier training stage, as shown in Fig 2a–2e, both orange and green bars are lower than other ones. This fact indicates better classifier training quality by using STRING2GO_Mashup+SVM_, STRING2GO_Node2vec+SVM_, STRING2GO_Mashup+Sigmoid_ and STRING2GO_Node2vec+Sigmoid_ than the ones obtained by Mashup+SVM and Node2vec+SVM, when using all five different STRING networks to generate embedding representations.

**Fig 3:**
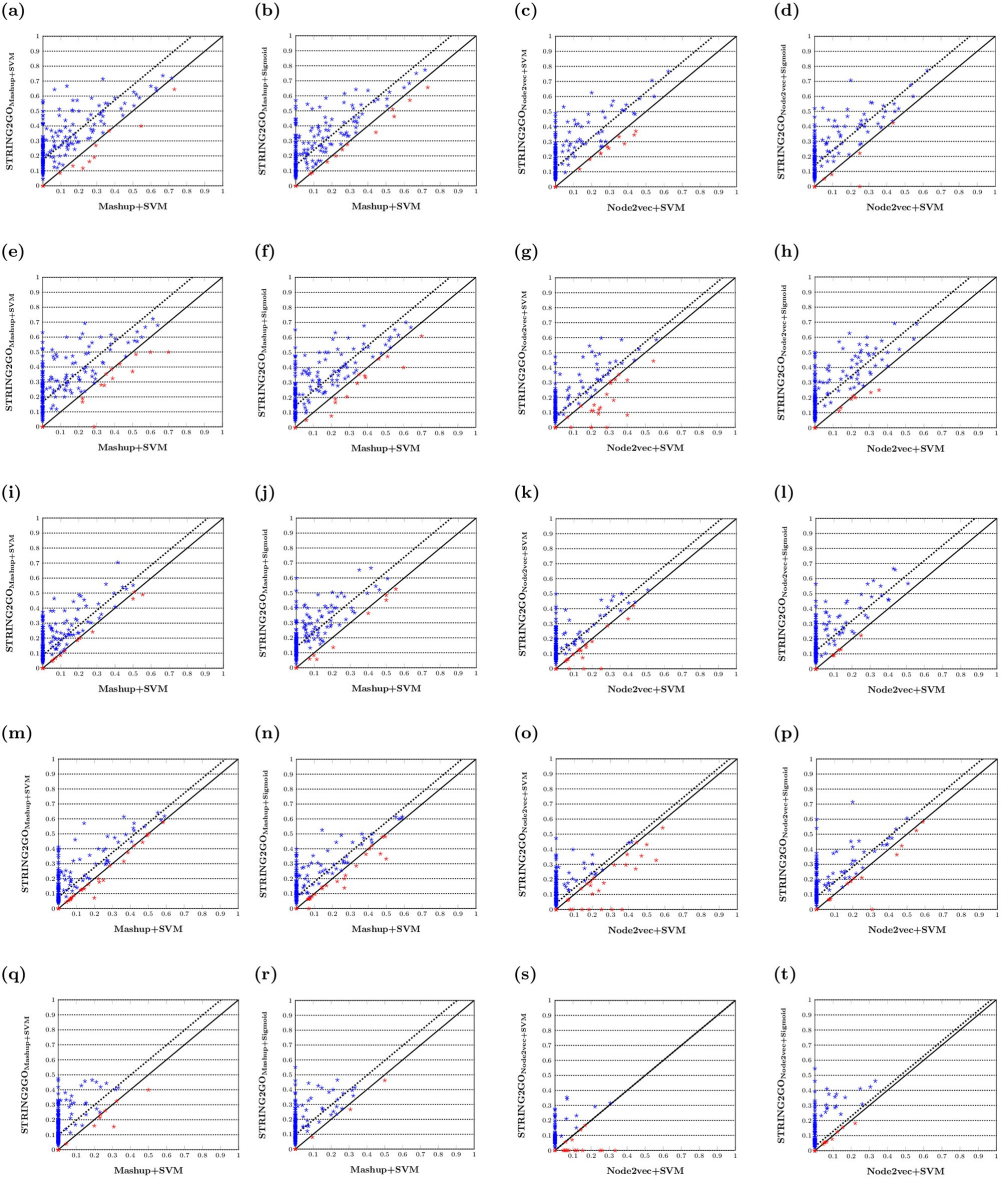
F_1_GO_ scores obtained by different network embedding representations and the corresponding STRING2GO-learnt functional representations based on (a-d) Combinedscore, (e-h) Textmining, (i-l) Experimental, (m-p) Database and (q-t) Coexpression networks by using SVM or Sigmoid function for classification.

The hold-out evaluation results further confirm that the STRING2GO-learnt functional representations contain higher predictive information. As shown in Table 2, the ${\tilde{F}}_{1\_\text{GO}}$ scores obtained by STRING2GO_Mashup+SVM_ and STRING2GO_Node2vec+SVM_ reach to 0.270 and 0.182 respectively, whereas the ${\tilde{F}}_{1\_\text{GO}}$ scores obtained by Mashup+SVM and Node2vec+SVM are both equal to 0.000. Analogously, the ${\tilde{\text{MCC}}}_{\text{GO}}$ scores obtained by STRING2GO_Mashup+SVM_ and STRING2GO_Node2vec+SVM_ reach 0.277 and 0.215 respectively. Both of them are higher than the zero ${\tilde{\text{MCC}}}_{\text{GO}}$ scores obtained by Mashup+SVM and Node2vec+SVM. This pattern is consistent when adopting all other types of STRING component networks, except STRING2GO_Node2vec+SVM_ and Node2vec+SVM both obtain zero ${\tilde{F}}_{1\_\text{GO}}$ and ${\tilde{\text{MCC}}}_{\text{GO}}$ scores when using the Coexpression network to generate the raw embedding representations (as shown in Table 2). In addition, both STRING2GO_Mashup+SVM_ and STRING2GO_Node2vec+SVM_ obtained higher ${\tilde{\text{AUPRC}}}_{\text{GO}}$ scores than Mashup+SVM and Node2vec+SVM methods based on all five different STRING networks. STRING2GO_Mashup+Sigmoid_ and STRING2GO_Node2vec+Sigmoid_ also respectively obtain higher ${\tilde{F}}_{1\_\text{GO}}$, ${\tilde{\text{MCC}}}_{\text{GO}}$ and ${\tilde{\text{AUPRC}}}_{\text{GO}}$ scores than Mashup+SVM and Node2vec+SVM based on all five different STRING networks. The scatter-plots in Fig 3 show the pairwise comparisons of F_1_GO_ scores obtained by different methods, and the dashed-lines indicate the median values of difference between pairs of F_1_GO_ scores. In detail, Fig 3a–3d show that almost all dots (in blue) drop in the area above the diagonal, indicating higher F_1_GO_ scores for predicting the majority of BP terms by using the functional representations learnt by STRING2GO based on the Combinedscore network by using either SVM or Sigmoid function as the classification algorithm. As shown in Fig 3e–3t, this pattern is consistently observed when applying on almost all other four different STRING networks, except the Coexpression network that leads to competitive performance between STRING2GO_Node2vec_ and Node2vec, since the dashed-lines in Fig 3s and 3t are almost overlapping on the diagonal. The Wilcoxon signed-rank test results in S5–S7 Tables further confirm that the STRING2GO-learnt functional representations obtain significantly higher GO term-centric F_1_GO_, MCC_GO_ and AUPRC_GO_ scores than the raw network embedding representations.

**Table 2:** Summary of GO term-centric results obtained by different network embedding representations and corresponding functional representations based on Combinedscore, Textmining, Experimental, Database and Coexpression networks working with different classification algorithms during hold-out evaluation.

Prediction Methods	Combinedscore			Textmining			Experimental			Database			Coexpression		
	${\tilde{F}}_{1\_\text{GO}}$	${\tilde{\text{MCC}}}_{\text{GO}}$	${\tilde{\text{AUPRC}}}_{\text{GO}}$	${\tilde{F}}_{1\_\text{GO}}$	${\tilde{\text{MCC}}}_{\text{GO}}$	${\tilde{\text{AUPRC}}}_{\text{GO}}$	${\tilde{F}}_{1\_\text{GO}}$	${\tilde{\text{MCC}}}_{\text{GO}}$	${\tilde{\text{AUPRC}}}_{\text{GO}}$	${\tilde{F}}_{1\_\text{GO}}$	${\tilde{\text{MCC}}}_{\text{GO}}$	${\tilde{\text{AUPRC}}}_{\text{GO}}$	${\tilde{F}}_{1\_\text{GO}}$	${\tilde{\text{MCC}}}_{\text{GO}}$	${\tilde{\text{AUPRC}}}_{\text{GO}}$
***Mashup-based***															
STRING2GO_Mashup+SVM_	**0.270**	**0.277**	0.226	**0.275**	0.263	0.210	0.146	0.174	0.157	0.130	0.161	0.127	0.116	0.142	0.106
STRING2GO_Mashup+Sigmoid_	0.237	0.261	0.232	0.239	**0.273**	**0.235**	**0.183**	**0.223**	**0.199**	**0.131**	0.164	**0.153**	**0.121**	**0.159**	**0.116**
Mashup+SVM	0.000	0.000	0.200	0.000	0.000	0.206	0.000	0.000	0.153	0.000	0.000	0.121	0.000	0.000	0.093
***Node2vec-based***															
STRING2GO_Node2ve+SVM_	0.182	0.215	0.190	0.115	0.169	0.191	0.124	0.174	0.160	0.087	0.135	0.129	0.000	0.000	0.084
STRING2GO_Node2vec+Sigmoid_	0.187	0.241	0.229	0.188	0.237	0.228	0.143	0.196	0.182	0.111	**0.168**	0.151	0.043	0.069	0.099
Node2vec+SVM	0.000	0.000	0.175	0.000	0.000	0.182	0.000	0.000	0.156	0.000	0.000	0.100	0.000	0.000	0.077

**Fig 4:**
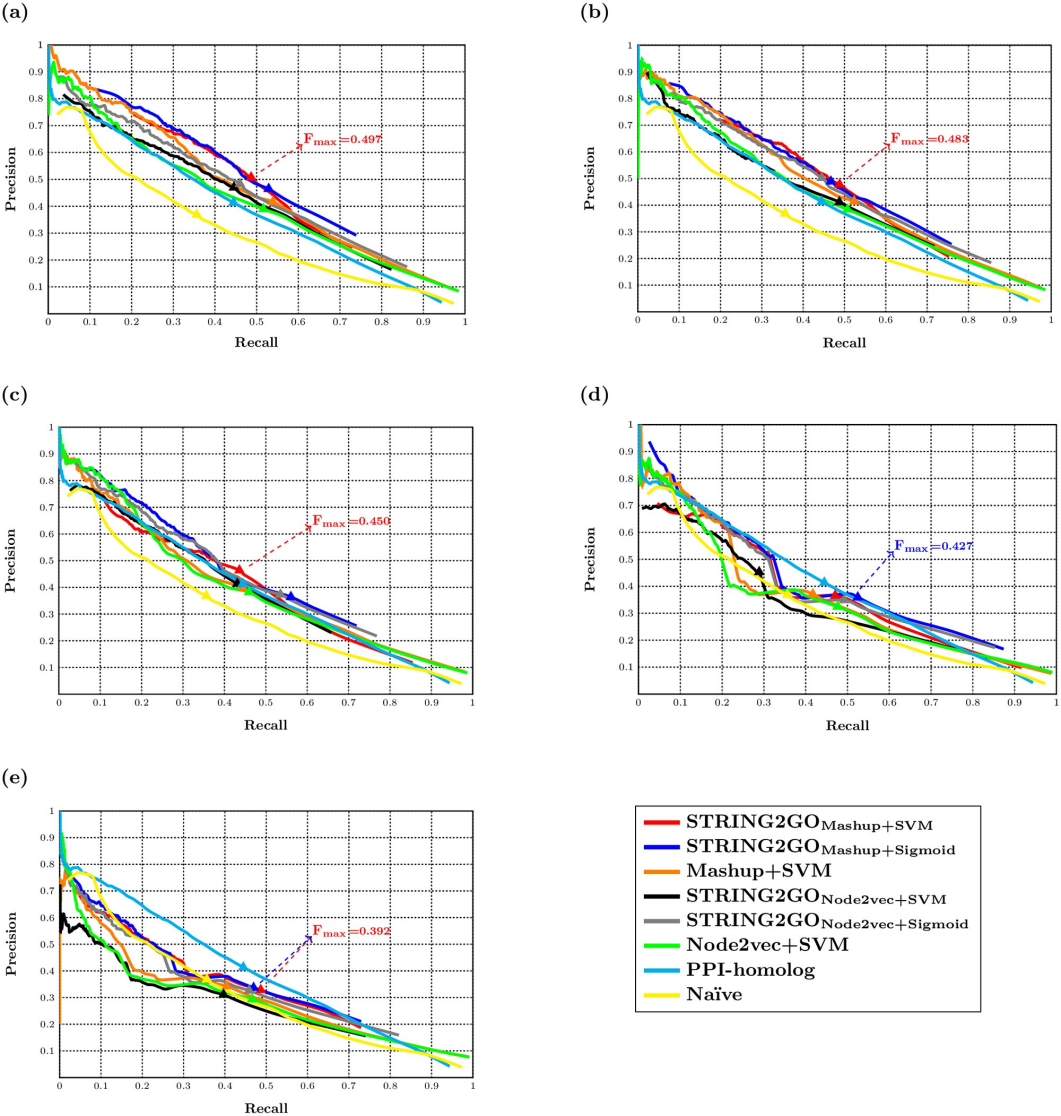
Precision-recall curves of different methods and the F_max_ scores obtained by the best-performing methods based on (a) Combinedscore, (b) Textmining, (c) Experimental, (d) Database and (e) Coexpression networks.

From the perspective of protein-centric evaluation (i.e. considering the F_max_ and F_*τ*_ metrics), the STRING2GO-learnt functional representations also obtain higher predictive accuracy based on the Combinedscore network. As shown in Table 3, the functional representations STRING2GO_Mashup_ and STRING2GO_Node2vec_ both obtain higher F_max_ scores (i.e. 0.497 and 0.458 obtained by using SVM, 0.495 and 0.471 obtained by using Sigmoid function) than the network embedding representations generated by Mashup and Node2vec (i.e. 0.470 and 0.444 obtained by using SVM). The precision-recall curves in Fig 4a also show that the STRING2GO-learnt functional representations obtain higher precision and recall values simultaneously, since the middle parts of red and blue curves locate in higher position than the orange one, while the middle parts of grey and black curves also locate in higher position than the green one. As shown in Table 3 and Fig 4b–4e, this pattern is consistent when adopting the other four types of STRING component networks to generate representations, except STRING2GO_Node2vec+SVM_ obtaining lower F_max_ scores than Node2vec+SVM based on the Database and Coexpression networks. In terms of the predictive performance on the homolog-removal hold-out sets with the E-value threshold of 10^−2^, STRING2GO_Mashup+SVM_ and STRING2GO_Mashup+Sigmoid_ both obtain higher F_max_ scores than Mashup+SVM over all STRING networks except the Coexpression network, while STRING2GO_Node2vec+Sigmoid_ also outperforms Node2vec+SVM over five STRING networks and STRING2GO_Node2vec+SVM_ obtains higher F_max_ score than Node2vec+SVM based on the Combinedscore network. In addition, all methods also obtain similar F_max_ scores over the evaluations on different degrees of homolog-removal hold-out sets, as reported in the S3 Table.

**Table 3:** Summary of protein-centric results obtained by different network embedding representations and corresponding functional representations based on Combinedscore, Textmining, Experimental, Database and Coexpression networks working with different classification algorithms during hold-out (Full) and homolog-removal (HR) hold-out evaluation, temporal (Full) and homolog-removal (HR) temporal validation.

Prediction Methods	Combinedscore				Textmining				Experimental				Database				Coexpression			
	F_max_		F_*τ*_		F_max_		F_*τ*_		F_max_		F_*τ*_		F_max_		F_*τ*_		F_max_		F_*τ*_	
	Full	HR	Full	HR	Full	HR	Full	HR	Full	HR	Full	HR	Full	HR	Full	HR	Full	HR	Full	HR
***Mashup-based***																				
STRING2GO_Mashup+SVM_	**0.497**	0.454	0.309	0.273	**0.483**	0.425	0.296	0.237	**0.450**	**0.412**	**0.263**	0.199	0.412	0.380	0.225	0.099	**0.392**	0.334	**0.258**	0.186
STRING2GO_Mashup+Sigmoid_	0.495	**0.464**	0.312	0.271	0.478	0.426	0.290	0.237	0.442	0.410	0.247	0.193	**0.427**	**0.409**	0.144	0.146	**0.392**	0.334	0.247	0.189
Mashup+SVM	0.470	0.427	0.290	0.249	0.463	0.411	0.287	0.231	0.420	0.358	0.229	0.166	0.392	0.373	**0.238**	0.186	0.371	0.336	0.242	0.199
***Node2vec-based***																				
STRING2GO_Node2ve+SVM_	0.458	0.423	**0.319**	0.280	0.446	0.398	0.290	0.288	0.422	0.370	0.256	0.215	0.353	0.351	0.169	**0.194**	0.349	0.307	0.236	0.216
STRING2GO_Node2vec+Sigmoid_	0.471	0.410	0.312	**0.284**	0.472	**0.428**	**0.314**	**0.298**	0.440	0.397	0.258	**0.224**	0.408	0.403	**0.238**	0.193	0.381	**0.356**	0.246	**0.232**
Node2vec+SVM	0.444	0.394	0.293	**0.284**	0.437	0.401	0.278	0.260	0.418	0.382	0.249	0.219	0.386	0.373	0.221	0.184	0.360	0.313	0.219	0.187
***Benchmark***																				
Naïve	0.363	0.342	0.254	0.223																
PPI-homolog	0.429	N/A*	0.363	N/A*																

* N/A: the results for the homolog-free hold-out and temporal validation protein-sets are not applicable since PPI-homolog method uses the homologous information to make predictions.

Analogously, the functional representations STRING2GO_Mashup_ and STRING2GO_Node2vec_ obtain higher F_*τ*_ scores based on the Combinedscroe network (0.309 and 0.319 obtained by SVM, while 0.312 obtained by Sigmoid function) than the raw network embedding representations generated by Mashup and Node2vec (0.290 and 0.293 by using SVM). This pattern is consistent when using all other STRING networks, except the Database network which only leads to higher F_*τ*_ score obtained by STRING2GO_Node2vec+Sigmoid_ than the one obtained by Node2vec+SVM. In terms of the predictive performance on the homolog-removal temporal validation protein-sets with an E-value threshold of 10^−2^, STRING2GO_Mashup+SVM_ and STRING2GO_Mashup+Sigmoid_ outperform Mashup+SVM based on the Combinedscore, Textmining and Experimental networks. Analogously, STRING2GO_Node2vec+Sigmoid_ obtains the same F_*τ*_ score to the Node2vec+SVM method based on the Combinedscore network and higher F_*τ*_ scores over all other four STRING networks. STRING2GO_Node2vec+SVM_ also outperforms Node2vec+SVM based on the Textmining, Database and Coexpression networks. The results obtained by all methods over the evaluations of different degrees of homolog-removal temporal validation sets are also similar.

### The raw network embedding representations derived by Mashup show higher predictive power

We also compare the predictive performance of Mashup and Node2vec-derived network embedding representations and the corresponding STRING2GO-learnt functional representations respectively. Generally, the raw network embedding representations derived by Mashup and Node2vec methods obtain competitive predictive accuracy by using SVM as the classification algorithm. To begin with, during the training stage, the ${\tilde{F}}_{1\_\text{GO}}$ score obtained by Mashup+SVM is higher than the one obtained by Node2vec+SVM based on the Combinedscore network, since the orange bar is higher than the green one in Fig 2a. However, both Mashup+SVM and Node2vec+SVM obtain poor predictive performance on the hold-out evaluation, due to the zero ${\tilde{F}}_{1\_\text{GO}}$ and ${\tilde{\text{MCC}}}_{\text{GO}}$ scores. But the statistical significance test results (see S5 and S6 Tables) show that the former still outperforms the latter. Those patterns are consistent when using all other 4 types of STRING networks to generate the raw embedding representations, except the fact that there is no significant difference on the MCC_GO_ scores obtained by the above two methods based on the Coexpression network, as reported in Fig 2b–2e, Table 2, S5 and S6 Tables. The Mashup+SVM method also obtains higher ${\tilde{\text{AUPRC}}}_{\text{GO}}$ scores and significantly higher AUPRC_GO_ scores over all 204 terms than Node2vec+SVM method based on four STRING networks, as reported in Table 2 and S7 Table. In terms of the protein-centric evaluation, Mashup+SVM obtains a higher F_max_ score (0.470) than Node2vec+SVM (0.444). The Combinedscore network-based precision-recall curves in Fig 4a confirm that the orange curve locates in higher position than the green one. Those patterns are also consistent in cases when using other four different STRING component networks to generate representations, as shown in Fig 4b–4e. Mashup+SVM also obtains higher F_max_ scores on the homolog-removal hold-out protein-sets based on the Combinedscore, Textmining and Coexpression networks. However, Node2vec+SVM outperforms Mashup+SVM on the temporal validation. As reported in Table 3, although the latter obtains higher F_*τ*_ score based on three STRING component networks (i.e. Textmining, Database and Coexpression), the former obtains the highest F_*τ*_ score (0.293) based on the Combinedscore network. Node2vec+SVM also obtains higher F_*τ*_ scores than Mashup+SVM on the homolog-removal temporal validation protein-sets based on the Combinedscore, Textmining and Experimental networks.

We then further conduct comparisons on predictive performance of two different STRING2GO-learnt functional representations respectively based on Mashup and Node2vec-derived raw network embedding representations. During the GO term-specific classifiers training stage, STRING2GO_Mashup_ obtains higher ${\tilde{F}}_{1\_\text{GO}}$ scores than STRING2GO_Node2vec_ by using either SVM or Sigmoid function as the classification algorithm, based on the Combinedscore and Coexpression networks. As shown in Fig 2a and 2e, where red and blue bars are higher than the black and grey ones respectively. When using the other 3 STRING component networks, STRING2GO_Node2vec_ obtains higher ${\tilde{F1}}_{\text{GO}}$ scores by using SVMs, whereas STRING2GO_Mashup_ still outperforms the former by using Sigmoid function as the classification algorithm.

The hold-out evaluation results in Tables 2 and 3 show a consistent pattern that STRING2GO_Mashup_ obtains higher ${\tilde{F}}_{1\_\text{GO}}$, ${\tilde{\text{MCC}}}_{\text{GO}}$ and ${\tilde{\text{AUPRC}}}_{\text{GO}}$ scores (statistically significant according to S5, S6 and S7 Tables) and F_max_ scores than STRING2GO_Node2vec_ based on the Combinedscore network by using either SVM or Sigmoid function, respectively. As shown in Fig 4a, the majority parts of the red and blue curves clearly locate in higher position than the black and grey ones. Those patterns are consistent when using the other 4 STRING networks, as shown in Table 3 and Fig 4b–4e. Analogously, STRING2GO_Mashup_ also obtains higher F_max_ scores than STRING2GO_Node2vec_ on the homolog-removal hold-out sets based on the Combinedscore, Experimental and Database networks by using either SVM or Sigmoid function, respectively. However, STRING2GO_Node2vec_ obtains better predictive performance during the temporal annotation validation, since the former obtains the highest F_*τ*_ score (0.319) by using SVM (based on the Combinedscore network) among all methods when adopting all different STRING networks. STRING2GO_Node2vec_ also obtains the overall highest F_*τ*_ score (0.298) on the homolog-removal temporal validation based on the Textmining network.

### The STRING2GO-learnt functional representations with support vector machines obtain the highest accuracy on predicting 204 BP terms

We then compare all prediction methods discussed in previous sections, i.e. two types of STRING2GO methods (i.e. STRING2GO_Embedding+SVM_ and STRING2GO_Embedding+Sigmoid_) adopting two types of raw network embedding representations (i.e. the ones generated by Mashup and Node2vec respectively), and the methods that only exploit the raw network embedding representations to make predictions by using SVM as the classification algorithm. We also compare those methods with the PPI-homolog [16] and Naïve prediction method [32]. The former makes predictions of target proteins’ GO term annotations by transferring corresponding annotations of PPI partners’ homologs defined by the BLAST search. The latter makes predictions by considering the annotation frequency in the database as the prior knowledge. Overall, STRING2GO_Embedding+SVM_ is the best-performing method according to both the GO term and protein-centric metrics. During the GO term-specific classifiers training stage, STRING2GO_Mashup+SVM_ and STRING2GO_Node2vec+SVM_ obtain almost the same highest ${\tilde{F}}_{1\_\text{GO}}$ scores among all prediction methods by using all different STRING networks. As shown in Fig 2, the latter obtains the highest ${\tilde{F}}_{1\_\text{GO}}$ score (0.824) based on the Textmining network, while the former obtained almost the same highest ${\tilde{F}}_{1\_\text{GO}}$ score (0.822) based on the Combinedscore network. The hold-out evaluation results also confirm that STRING2GO_Mashup+SVM_ obtains the highest ${\tilde{F}}_{1\_\text{GO}}$ score (0.275) by using the Textmining network, while also obtains the significantly higher F_1_GO_ scores than other methods basing on the Combinescore network (see the Friedman test with Holm *post-hoc* correction results in S8 Table). STRING2GO_Mashup+SVM_ also obtains the overall highest MCC_GO_ score (0.277) based on the Combinedscore network and significantly higher MCC_GO_ scores over all 204 GO terms than all other methods based on the Textmining network (see the Friedman test with Holm *post-hoc* correction results in S9 Table). In terms of the protein-centric evaluation metrics, STRING2GO_Mashup+SVM_ obtains the highest F_max_ score (0.497) based on the Combinedscore network and higher F_max_ scores than all other methods based on all other STRING networks except the Database network. It also obtains the second highest F_max_ score on the homolog-removal hold-out evaluation protein-set based on the Combinedscore network. In terms of the F_*τ*_ score metric, STRING2GO_Node2vec+SVM_ obtains the highest F_*τ*_ score (0.319) by using the Combinedscore network among all network embedding-based prediction methods based on all different STRING networks.

The second best performing method is STRING2GO_Embedding+Sigmoid_. STRING2GO_Mashup+Sigmoid_ obtains higher ${\tilde{F}}_{1\_\text{GO}}$ scores than either Mashup+SVM or Node2vec+SVM during the classifier training stage. It also obtains the second highest ${\tilde{F}}_{1\_\text{GO}}$ scores during the hold-out evaluation based on 2 out of 5 networks (except the case when STRING2GO_Mashup+Sigmoid_ obtains the highest ${\tilde{F}}_{1\_\text{GO}}$ score based on the Experimental, Database and Coexpression networks). It also obtains the overall second highest ${\tilde{\text{MCC}}}_{\text{GO}}$ score (0.273) based on the Textmining network. In terms of the AUPRC metric, STRING2GO_Mashup+Sigmoid_ obtains the overall highest ${\tilde{\text{AUPRC}}}_{\text{GO}}$ score (0.235) based on the Textmining network, and significantly higher AUPRC scores over all 204 BP terms than other methods based on the Combinedscore, Textmining and Experimental networks (see the Friedman test with Holm *post-hoc* correction results in S10 Table). From the perspective of protein-centric metrics, STRING2GO_Mashup+Sigmoid_ obtains the second highest F_max_ based on 3 out of 5 STRING networks, and the highest F_max_ score (0.464) based on the homolog-removal hold-out set with the Combinedscore network. Analogously, STRING2GO_Node2vec+Sigmoid_ also obtains the overall highest F_*τ*_ score (0.298) over all prediction methods based on the homolog-removal temporal validation protein-set with the Textmining network.

In addition, all of those methods discussed above obtains higher F_max_ scores than the PPI-homolog and Naïve prediction methods based on the Combinedscore and Textmining networks. All those methods also obtain higher F_*τ*_ scores than the Naïve prediction method based on the Combinedscore and Textmining networks, whereas the PPI-homolog method obtains the overall highest F_*τ*_ score (0.363).

## Discussion

**Fig 5:**
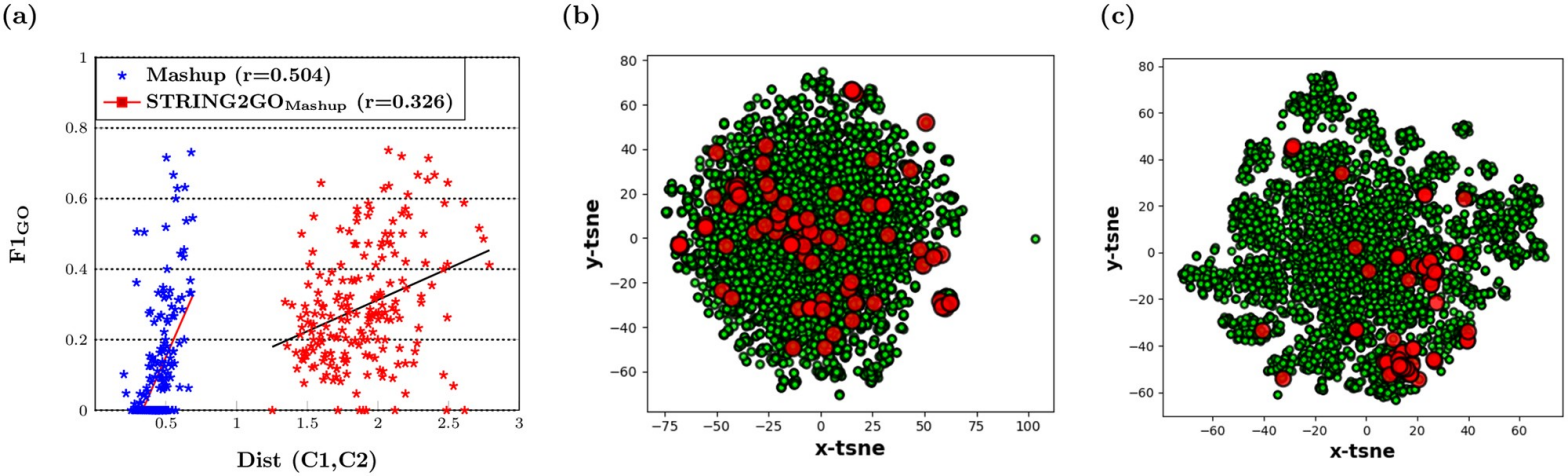
(a) Linear relationship between distances of two classes protein samples and F_1_GO_ scores obtained by Mashup-derived Combinedscore network embedding representations and the corresponding functional representations on Hold-out set testing (b) The 2D space visualization of distribution of protein samples belonging to GO:0090150 using the Mashup-derived network embedding representations and (c) the STRING2GO_Mashup_ functional representations transformed by t-SNE.

Overall, as discussed in previous sections, the functional representations learnt by STRING2GO show substantial improvement on the predictive power of the raw network embedding representations. We further investigate the improvement of predictive power of the STRING2GO-learnt functional representations by evaluating the enlarged distances between two classes of training protein samples. We firstly calculate the Euclidean distance between the centroids of two classes by using the Mashup-based representations’ values standardized into the range of (0,1) in the same dimensional space, i.e. 800 dimensions for both Mashup and STRING2GO_Mashup_. Then we calculate the correlation coefficient between the distances and F_1_GO_ scores obtained by hold-out evaluation. As shown in Fig 5a, the *x* axis denotes the distance between two classes calculated by using either the raw Mashup-derived network embedding representations (blue), or the corresponding functional representations (red) STRING2GO_Mashup_, based on the Combinedscore network, while the *y* axis denotes the corresponding F_1_GO_ score obtained by adopting those different representations working with SVMs to predict individual BP terms. It is obvious that the distances between two classes of proteins for individual GO terms are all enlarged by STRING2GO, while the correlation coefficient values between distances and F_1_GO_ scores for both types of representations are positive, indicating that the larger distances lead to higher predictive accuracy.

We also display an example of the increased distance between two classes of proteins when predicting the term GO:0090150, which shows the highest improvement on the classifier training quality obtained by using STRING2GO_Mashup+SVM_, compared by using Mashup+SVM. Fig 5b and 5c respectively show the 2-D visualization of raw Mashup-derived network embedding representations and the corresponding STRING2GO-learnt functional representations after transforming by t-SNE [42]. The red dots denote the protein samples belonging to class “Annotated”, while the green dots denote the protein samples belonging to class “Not-annotated”. The red dots are distributed in the similar scale of green dots in Fig 5b, whereas the most of red dots are clustered in the right side in Fig 5c. This fact indicates that the functional representations successfully encode higher discriminating power against two classes of protein samples.

## Conclusion

In this work, we present a novel deep learning-based protein function prediction method STRING2GO, which successfully learns a novel type of functional representations to train the down-stream classifiers for making predictions. STRING2GO shows the highest accuracy when predicting biological process protein functions, compared with other state-of-the-art network embedding representation-based protein function prediction methods. Based on this STRING2GO learning framework, there is potential for further improving the predictive accuracy by integrating representations from other data sources with the current PPI network embedding representations in a future study.

## Supporting information

S1 Table List of 204 biological process Gene Ontology terms studied in this work. (XLS)

S2 Table Summary of number of proteins in the homolog-removal hold-out and temporal hold-out protein-sets after applying different E-value thresholds of the BLAST search. (PDF)

S3 Table Summary of F_max_ scores obtained by different degrees of homolog-removal hold-out protein-sets obtained by using different prediction methods. (PDF)

S4 Table Summary of F_*τ*_ scores obtained by different degrees of homolog-removal temporal validation protein-sets obtained by using different prediction methods. (PDF)

S5 Table Two-tailed Wilcoxon signed-rank test results at the significance level of 0.05 on F_1_GO_ scores obtained by different pairs of prediction methods over the hold-out evaluation. (PDF)

S6 Table Two-tailed Wilcoxon signed-rank test results at the significance level of 0.05 on MCC_GO_ scores obtained by different pairs of prediction methods over the hold-out evaluation. (PDF)

S7 Table Two-tailed Wilcoxon signed-rank test results at the significance level of 0.05 on AUPRC_GO_ scores obtained by different pairs of prediction methods over the hold-out evaluation. (PDF)

S8 Table Friedman test with the Holm *post-hoc* correction results about multiple comparisons on F_1_GO_ scores obtained by different prediction methods over the hold-out evaluation. (PDF)

S9 Table Friedman test with the Holm *post-hoc* correction results about multiple comparisons on MCC_GO_ scores obtained by different prediction methods over the hold-out evaluation. (PDF)

S10 Table Friedman test with the Holm *post-hoc* correction results about multiple comparisons on AUPRC_GO_ scores obtained by different prediction methods over the hold-out evaluation. (PDF)
