# Supplementary material for: Using deep maxout neural networks to improve the accuracy of function prediction from protein interaction networks
Source: PLoS One. 2019 Jul 23;14(7):e0209958. doi: 10.1371/journal.pone.0209958 (PMC6650051; doi:10.1371/journal.pone.0209958)
Supplement: S2 Table — (PDF) [file pone.0209958.s002.pdf]

S2 Table. Summary of number of proteins in the homolog-removal hold-out and temporal hold-out protein-sets after applying different E-value thresholds of the BLAST search.

E-value threshold	E-05	E-04	E-03	E-02
Hold-out protein-sets				
Number of Proteins	255	240	220	192
Temporal annotation validation protein-sets				
Number of Proteins	198	193	191	182
