# Supplementary material for: Using deep maxout neural networks to improve the accuracy of function prediction from protein interaction networks
Source: PLoS One. 2019 Jul 23;14(7):e0209958. doi: 10.1371/journal.pone.0209958 (PMC6650051; doi:10.1371/journal.pone.0209958)
Supplement: S3 Table — (PDF) [file pone.0209958.s003.pdf]

S3 Table. Summary of F_{max} scores obtained by different degrees of homolog-removal hold-out protein-sets obtained by using different prediction methods.

E-value threshold	E-05	E-04	E-03	E-02
Combinescore network				
Mashup+MDNN+SVM	0.471	0.468	0.459	0.454
Mashup+MDNN	0.480	0.476	0.470	0.464
Mashup+SVM	0.444	0.441	0.433	0.427
Node2vec+MDNN+SVM	0.438	0.436	0.431	0.423
Node2vec +MDNN	0.430	0.420	0.417	0.410
Node2vec +SVM	0.408	0.402	0.401	0.394
Textmining network				
Mashup+MDNN+SVM	0.445	0.442	0.431	0.425
Mashup+MDNN	0.447	0.445	0.436	0.426
Mashup+SVM	0.428	0.424	0.416	0.411
Node2vec+MDNN+SVM	0.415	0.408	0.406	0.398
Node2vec +MDNN	0.449	0.440	0.435	0.428
Node2vec +SVM	0.417	0.413	0.409	0.401
Experimental network				
Mashup+MDNN+SVM	0.423	0.420	0.422	0.412
Mashup+MDNN	0.420	0.413	0.410	0.410
Mashup+SVM	0.372	0.368	0.364	0.358
Node2vec+MDNN+SVM	0.374	0.375	0.371	0.370
Node2vec +MDNN	0.402	0.400	0.399	0.397
Node2vec +SVM	0.387	0.382	0.380	0.382
Database network				
Mashup+MDNN+SVM	0.400	0.393	0.386	0.380
Mashup+MDNN	0.420	0.417	0.407	0.409
Mashup+SVM	0.381	0.380	0.375	0.373
Node2vec+MDNN+SVM	0.354	0.348	0.349	0.351
Node2vec +MDNN	0.404	0.403	0.398	0.403
Node2vec +SVM	0.381	0.380	0.375	0.373
Coexpression network				
Mashup+MDNN+SVM	0.345	0.336	0.331	0.334
Mashup+MDNN	0.347	0.343	0.332	0.334
Mashup+SVM	0.331	0.333	0.335	0.336
Node2vec+MDNN+SVM	0.313	0.307	0.300	0.307
Node2vec +MDNN	0.371	0.367	0.358	0.356
Node2vec +SVM	0.321	0.316	0.313	0.313
Benchmark method				
Naive	0.347	0.345	0.344	0.342
