# Supplementary material for: Using deep maxout neural networks to improve the accuracy of function prediction from protein interaction networks
Source: PLoS One. 2019 Jul 23;14(7):e0209958. doi: 10.1371/journal.pone.0209958 (PMC6650051; doi:10.1371/journal.pone.0209958)
Supplement: S4 Table — (PDF) [file pone.0209958.s004.pdf]

S4 Table. Summary of Fτ scores obtained by different degrees of homolog-removal temporal validation protein-sets obtained by using different prediction methods.

E-value threshold	E-05	E-04	E-03	E-02
Combinedscore network				
Mashup+MDNN+SVM	0.265	0.271	0.264	0.273
Mashup+MDNN	0.253	0.256	0.258	0.271
Mashup+SVM	0.239	0.241	0.243	0.249
Node2vec+MDNN+SVM	0.269	0.272	0.274	0.280
Node2vec +MDNN	0.288	0.291	0.282	0.284
Node2vec +SVM	0.275	0.277	0.278	0.284
Textmining network				
Mashup+MDNN+SVM	0.227	0.230	0.231	0.237
Mashup+MDNN	0.237	0.238	0.232	0.237
Mashup+SVM	0.225	0.229	0.230	0.231
Node2vec+MDNN+SVM	0.276	0.280	0.282	0.288
Node2vec +MDNN	0.279	0.291	0.288	0.298
Node2vec +SVM	0.250	0.254	0.257	0.260
Experimental network				
Mashup+MDNN+SVM	0.190	0.191	0.192	0.199
Mashup+MDNN	0.186	0.188	0.187	0.193
Mashup+SVM	0.164	0.165	0.164	0.166
Node2vec+MDNN+SVM	0.210	0.212	0.210	0.215
Node2vec +MDNN	0.219	0.223	0.220	0.224
Node2vec +SVM	0.213	0.214	0.214	0.219
Database network				
Mashup+MDNN+SVM	0.096	0.095	0.096	0.099
Mashup+MDNN	0.143	0.144	0.145	0.146
Mashup+SVM	0.180	0.183	0.183	0.186
Node2vec+MDNN+SVM	0.189	0.187	0.188	0.194
Node2vec +MDNN	0.186	0.188	0.188	0.193
Node2vec +SVM	0.177	0.179	0.179	0.184
Coexpression network				
Mashup+MDNN+SVM	0.185	0.182	0.183	0.186
Mashup+MDNN	0.188	0.187	0.188	0.189
Mashup+SVM	0.194	0.196	0.196	0.199
Node2vec+MDNN+SVM	0.209	0.211	0.212	0.216
Node2vec +MDNN	0.232	0.232	0.232	0.232
Node2vec +SVM	0.184	0.184	0.185	0.187
Benchmark method				
Naive	0.220	0.221	0.220	0.223
