# Supplementary material for: Using deep maxout neural networks to improve the accuracy of function prediction from protein interaction networks
Source: PLoS One. 2019 Jul 23;14(7):e0209958. doi: 10.1371/journal.pone.0209958 (PMC6650051; doi:10.1371/journal.pone.0209958)
Supplement: S5 Table — (PDF) [file pone.0209958.s005.pdf]

**S5 Table.** Two-tailed Wilcoxon signed-rank test results at the significance level of 0.05 on $F_{1\_GO}$ scores obtained by different pairs of prediction methods over the hold-out evaluation.

| | Methods | Combinedscore | Textmining | Experimental | Database | Coexpression |
|---|---|---|---|---|---|---|
| 1 | $STRING2GO_{Mashup+SVM}$ (**ctrl.**)<br>Mashup+SVM | +<br>(< 2.2e-16) | +<br>(< 2.2e-16) | +<br>(< 2.2e-16) | +<br>(< 2.2e-16) | +<br>(< 2.2e-16) |
| 2 | $STRING2GO_{Node2vec+SVM}$ (**ctrl.**)<br>Node2vec+SVM | +<br>(< 2.2e-16) | +<br>(< 2.2e-16) | +<br>(< 2.2e-16) | +<br>(1.8e-13) | +<br>(1.4e-08) |
| 3 | $STRING2GO_{Mashup+Sigmoid}$ (**ctrl.**)<br>Mashup+SVM | +<br>(< 2.2e-16) | +<br>(< 2.2e-16) | +<br>(< 2.2e-16) | +<br>(< 2.2e-16) | +<br>(< 2.2e-16) |
| 4 | $STRING2GO_{Node2vec+Sigmoid}$ (**ctrl.**)<br>Node2vec+SVM | +<br>(< 2.2e-16) | +<br>(< 2.2e-16) | +<br>(< 2.2e-16) | +<br>(< 2.2e-16) | +<br>(< 2.2e-16) |
| 5 | Mashup+SVM (**ctrl.**)<br>Node2ve+SVM | +<br>(1.1e-10) | +<br>(2.3e-07) | +<br>(4.2e-05) | +<br>(7.2e-05) | +<br>(1.6e-02) |
| 6 | $STRING2GO_{Mashup+SVM}$ (**ctrl.**)<br>$STRING2GO_{Node2vec+SVM}$ | +<br>(< 2.2e-16) | +<br>(< 2.2e-16) | +<br>(3.7e-04) | +<br>(3.8e-10) | +<br>(< 2.2e-16) |
| 7 | $STRING2GO_{Mashup+Sigmoid}$ (**ctrl.**)<br>$STRING2GO_{Node2vec+Sigmoid}$ | +<br>(1.4e-14) | +<br>(< 2.2e-16) | +<br>(7.2e-05) | +<br>(2.2e-03) | +<br>(2.1e-11) |

+ (*p-value*): the control (ctrl.) method significantly outperforms the counterpart.
