# Supplementary material for: Using deep maxout neural networks to improve the accuracy of function prediction from protein interaction networks
Source: PLoS One. 2019 Jul 23;14(7):e0209958. doi: 10.1371/journal.pone.0209958 (PMC6650051; doi:10.1371/journal.pone.0209958)
Supplement: S6 Table — (PDF) [file pone.0209958.s006.pdf]

S6 Table. Two-tailed Wilcoxon signed-rank test results at the significance level of 0.05 on MCC_{GO} scores obtained by different pairs of prediction methods over the hold-out evaluation.

Methods	Combinedscore	Textmining	Experimental	Database	Coexpression
$STRING2GO_{Mashup+SVM}$ **(ctrl.)**<br>Mashup+SVM	+<br>(< 2.2e-16)	+<br>(< 2.2e-16)	+<br>(< 2.2e-16)	+<br>(< 2.2e-16)	+<br>(< 2.2e-16)
$STRING2GO_{Node2vec+SVM}$ **(ctrl.)**<br>Node2vec+SVM	+<br>(< 2.2e-16)	+<br>(< 2.2e-16)	+<br>(< 2.2e-16)	+<br>(8.2e-12)	+<br>(5.2e-05)
$STRING2GO_{Mashup+Sigmoid}$ **(ctrl.)**<br>Mashup+SVM	+<br>(< 2.2e-16)	+<br>(< 2.2e-16)	+<br>(< 2.2e-16)	+<br>(< 2.2e-16)	+<br>(< 2.2e-16)
$STRING2GO_{Node2vec+Sigmoid}$ **(ctrl.)**<br>Node2vec+SVM	+<br>(< 2.2e-16)	+<br>(< 2.2e-16)	+<br>(< 2.2e-16)	+<br>(< 2.2e-16)	+<br>(< 2.2e-16)
Mashup+SVM **(ctrl.)**<br>Node2ve+SVM	+<br>(1.1e-09)	+<br>(6.9e-07)	+<br>(2.3e-04)	+<br>(5.8e-05)	Ø<br>(8.4e-02)
$STRING2GO_{Mashup+SVM}$ **(ctrl.)**<br>$STRING2GO_{Node2vec+SVM}$	+<br>(1.0e-13)	+<br>(< 2.2e-16)	Ø<br>(1.6e-01)	+<br>(2.3e-06)	+<br>(< 2.2e-16)
$STRING2GO_{Mashup+Sigmoid}$ **(ctrl.)**<br>$STRING2GO_{Node2vec+Sigmoid}$	+<br>(8.8e-09)	+<br>(6.1e-06)	+<br>(2.6e-03)	Ø<br>(2.5e-01)	+<br>(2.4e-06)

+ (*p-value*): the control (ctrl.) method significantly outperforms the counterpart.
