# Supplementary material for: Using deep maxout neural networks to improve the accuracy of function prediction from protein interaction networks
Source: PLoS One. 2019 Jul 23;14(7):e0209958. doi: 10.1371/journal.pone.0209958 (PMC6650051; doi:10.1371/journal.pone.0209958)
Supplement: S7 Table — (PDF) [file pone.0209958.s007.pdf]

S7 Table. Two-tailed Wilcoxon signed-rank test results at the significance level of 0.05 on AUPRC$_{\text{GO}}$ scores obtained by different pairs of prediction methods over the hold-out evaluation.

Methods	Combinedscore	Textmining	Experimental	Database	Coexpression
STRING2GO$_{\text{Mashup+SVM}}$ **(ctrl.)**	+	∅	+	∅	+
Mashup+SVM	(3.9e-03)	(7.1e-01)	(3.7e-02)	(1.8e-01)	(5.0e-07)
STRING2GO$_{\text{Node2vec+SVM}}$ **(ctrl.)**	∅	+	∅	+	∅
Node2vec+SVM	(6.1e-01)	(8.9e-03)	(5.6e-01)	(4.1e-02)	(3.0e-01)
STRING2GO$_{\text{Mashup+Sigmoid}}$ **(ctrl.)**	+	+	+	+	+
Mashup+SVM	(1.3e-08)	(1.9e-08)	(1.3e-19)	(2.6e-11)	(1.6e-12)
STRING2GO$_{\text{Node2vec+Sigmoid}}$ **(ctrl.)**	+	+	+	+	+
Node2vec+SVM	(4.0e-11)	(5.8e-13)	(2.5e-09)	(7.8e-16)	(1.1e-11)
Mashup+SVM **(ctrl.)**	+	+	∅	+	+
Node2ve+SVM	(4.6e-06)	(2.3e-04)	(1.0e-01)	(2.8e-05)	(2.5e-03)
STRING2GO$_{\text{Mashup+SVM}}$ **(ctrl.)**	+	+	∅	+	+
STRING2GO$_{\text{Node2vec+SVM}}$	(3.4e-10)	(3.2e-04)	(7.5e-01)	(2.3e-03)	(3.7e-10)
STRING2GO$_{\text{Mashup+Sigmoid}}$ **(ctrl.)**	+	+	+	+	+
STRING2GO$_{\text{Node2vec+Sigmoid}}$	(4.0e-05)	(2.5e-03)	(2.0e-03)	(4.9e-02)	(2.1e-02)
+ (*p-value*): the control (ctrl.) method significantly outperforms the counterpart.					
