# Supplementary material for: Using deep maxout neural networks to improve the accuracy of function prediction from protein interaction networks
Source: PLoS One. 2019 Jul 23;14(7):e0209958. doi: 10.1371/journal.pone.0209958 (PMC6650051; doi:10.1371/journal.pone.0209958)
Supplement: S8 Table — (PDF) [file pone.0209958.s008.pdf]

S8 Table. Friedman test with the Holm *post-hoc* correction results about multiple comparisons on F_{1_GO} scores obtained by different prediction methods over the hold-out evaluation.

Combinedscore			
Methods	Average Rank	P-value	Adjusted α
STRING2GO$_{Mashup+SVM}$ (ctrl.)	1.86	N/A	N/A
STRING2GO$_{Mashup+Sigmoid}$	2.31	1.5e-02	5.0e-2
STRING2GO$_{Node2vec+Sigmoid}$	3.32	3.0e-15	2.5e-2
STRING2GO$_{Node2vec+SVM}$	3.39	1.3e-16	1.7e-2
Mashup+SVM	4.76	<2.2e-16	1.3e-2
Node2ve+SVM	5.35	<2.2e-16	1.0e-2

Textmining			
Methods	Average Rank	P-value	Adjusted α
STRING2GO$_{Mashup+SVM}$ (ctrl.)	1.88	N/A	N/A
STRING2GO$_{Mashup+Sigmoid}$	2.24	5.1e-02	5.0e-2
STRING2GO$_{Node2vec+Sigmoid}$	2.98	2.7e-09	2.5e-2
STRING2GO$_{Node2vec+SVM}$	3.99	<2.2e-16	1.7e-2
Mashup+SVM	4.70	<2.2e-16	1.3e-2
Node2ve+SVM	5.22	<2.2e-16	1.0e-2

Experimental			
Methods	Average Rank	P-value	Adjusted α
STRING2GO$_{Mashup+Sigmoid}$ (ctrl.)	2.26	N/A	N/A
STRING2GO$_{Node2vec+Sigmoid}$	2.70	1.7e-02	5.0e-2
STRING2GO$_{Mashup+SVM}$	2.85	1.4e-03	2.5e-2
STRING2GO$_{Node2vec+SVM}$	3.29	2.5e-08	1.7e-2
Mashup+SVM	4.80	<2.2e-16	1.3e-2
Node2ve+SVM	5.11	<2.2e-16	1.0e-2

Database			
Methods	Average Rank	P-value	Adjusted α
STRING2GO$_{Mashup+SVM}$ (ctrl.)	2.54	N/A	N/A
STRING2GO$_{Mashup+Sigmoid}$	2.63	6.3e-01	5.0e-2
STRING2GO$_{Node2vec+Sigmoid}$	2.96	2.3e-02	2.5e-2
STRING2GO$_{Node2vec+SVM}$	3.49	2.7e-07	1.7e-2
Mashup+SVM	4.50	<2.2e-16	1.3e-2
Node2ve+SVM	4.88	<2.2e-16	1.0e-2

Coexpression			
Methods	Average Rank	P-value	Adjusted α
STRING2GO$_{Mashup+SVM}$ (ctrl.)	2.13	N/A	N/A
STRING2GO$_{Mashup+Sigmoid}$	2.29	3.9e-01	5.0e-2
STRING2GO$_{Node2vec+Sigmoid}$	3.25	1.4e-09	2.5e-2
STRING2GO$_{Node2vec+SVM}$	4.01	<2.2e-16	1.7e-2
Mashup+SVM	4.59	<2.2e-16	1.3e-2
Node2ve+SVM	4.73	<2.2e-16	1.0e-2
