# Supplementary material for: Using deep maxout neural networks to improve the accuracy of function prediction from protein interaction networks
Source: PLoS One. 2019 Jul 23;14(7):e0209958. doi: 10.1371/journal.pone.0209958 (PMC6650051; doi:10.1371/journal.pone.0209958)
Supplement: S9 Table — (PDF) [file pone.0209958.s009.pdf]

**S9 Table.** Friedman test with the Holm *post-hoc* correction results about multiple comparisons on $MCC_{GO}$ scores obtained by different prediction methods over the hold-out evaluation.

| Combinedscore | | | | Textmining | | | |
|---|---|---|---|---|---|---|---|
| Methods | Average Rank | P-value | Adjusted α | Methods | Average Rank | P-value | Adjusted α |
| STRING2GO$_{\text{Mashup+SVM}}$ (ctrl.) | 2.34 | N/A | N/A | STRING2GO$_{\text{Mashup+SVM}}$ (ctrl.) | 2.38 | N/A | N/A |
| STRING2GO$_{\text{Mashup+Sigmoid}}$ | 2.50 | 3.9e-1 | 5.0e-2 | STRING2GO$_{\text{Mashup+Sigmoid}}$ | 2.89 | 5.8e-3 | 5.0e-2 |
| STRING2GO$_{\text{Node2vec+Sigmoid}}$ | 3.23 | 1.5e-6 | 2.5e-2 | STRING2GO$_{\text{Node2vec+Sigmoid}}$ | 3.14 | 4.0e-5 | 2.5e-2 |
| STRING2GO$_{\text{Node2vec+SVM}}$ | 3.49 | 5.0e-10 | 1.7e-2 | STRING2GO$_{\text{Node2vec+SVM}}$ | 3.68 | 2.1e-12 | 1.7e-2 |
| Mashup+SVM | 4.38 | <2.2e-16 | 1.3e-2 | Mashup+SVM | 4.44 | <2.2e-16 | 1.3e-2 |
| Node2ve+SVM | 5.06 | <2.2e-16 | 1.0e-2 | Node2ve+SVM | 5.22 | <2.2e-16 | 1.0e-2 |

| Experimental | | | | Database | | | |
|---|---|---|---|---|---|---|---|
| Methods | Average Rank | P-value | Adjusted α | Methods | Average Rank | P-value | Adjusted α |
| STRING2GO$_{\text{Mashup+Sigmoid}}$ (ctrl.) | 2.74 | N/A | N/A | STRING2GO$_{\text{Mashup+SVM}}$ (ctrl.) | 2.83 | N/A | N/A |
| STRING2GO$_{\text{Node2vec+Sigmoid}}$ | 2.94 | 2.8e-1 | 5.0e-2 | STRING2GO$_{\text{Mashup+Sigmoid}}$ | 3.00 | 3.6e-1 | 5.0e-2 |
| STRING2GO$_{\text{Mashup+SVM}}$ | 3.05 | 9.3e-2 | 2.5e-2 | STRING2GO$_{\text{Node2vec+Sigmoid}}$ | 3.14 | 9.3e-2 | 2.5e-2 |
| STRING2GO$_{\text{Node2vec+SVM}}$ | 3.18 | 1.7e-2 | 1.7e-2 | STRING2GO$_{\text{Node2vec+SVM}}$ | 3.49 | 3.6e-4 | 1.7e-2 |
| Mashup+SVM | 4.23 | 8.3e-16 | 1.3e-2 | Mashup+SVM | 4.07 | 2.1e-11 | 1.3e-2 |
| Node2ve+SVM | 5.28 | <2.2e-16 | 1.0e-2 | Node2ve+SVM | 4.46 | <2.2e-16 | 1.0e-2 |

| Coexpression | | | |
|---|---|---|---|
| Methods | Average Rank | P-value | Adjusted α |
| STRING2GO$_{\text{Mashup+SVM}}$ (ctrl.) | 2.64 | N/A | N/A |
| STRING2GO$_{\text{Mashup+Sigmoid}}$ | 2.71 | 7.1e-1 | 5.0e-2 |
| STRING2GO$_{\text{Node2vec+Sigmoid}}$ | 3.25 | 9.7e-4 | 2.5e-2 |
| STRING2GO$_{\text{Node2vec+SVM}}$ | 3.98 | 4.5e-13 | 1.7e-2 |
| Mashup+SVM | 4.17 | <2.2e-16 | 1.3e-2 |
| Node2ve+SVM | 4.25 | <2.2e-16 | 1.0e-2 |
