# Supplementary material for: Using deep maxout neural networks to improve the accuracy of function prediction from protein interaction networks
Source: PLoS One. 2019 Jul 23;14(7):e0209958. doi: 10.1371/journal.pone.0209958 (PMC6650051; doi:10.1371/journal.pone.0209958)
Supplement: S10 Table — (PDF) [file pone.0209958.s010.pdf]

S10 Table. Friedman test with the Holm *post-hoc* correction results about multiple comparisons on AUPRC$_{GO}$ scores obtained by different prediction methods over the hold-out evaluation.

Combinedscore			
Methods	Average Rank	P-value	Adjusted α
STRING2GO$_{\text{Mashup+Sigmoid}}$ (ctrl.)	2.63	N/A	N/A
STRING2GO$_{\text{Node2vec+Sigmoid}}$	3.09	1.3e-2	5.0e-2
STRING2GO$_{\text{Mashup+SVM}}$	3.14	5.8e-3	2.5e-2
Mashup+SVM	3.65	3.6e-8	1.7e-2
STRING2GO$_{\text{Node2vec+SVM}}$	4.20	<2.2e-16	1.3e-2
Node2ve+SVM	4.27	<2.2e-16	1.0e-2

Textmining			
Methods	Average Rank	P-value	Adjusted α
STRING2GO$_{\text{Mashup+Sigmoid}}$ (ctrl.)	2.58	N/A	N/A
STRING2GO$_{\text{Node2vec+Sigmoid}}$	3.01	2.0e-2	5.0e-2
STRING2GO$_{\text{Mashup+SVM}}$	3.46	1.9e-6	2.5e-2
Mashup+SVM	3.60	3.6e-8	1.7e-2
STRING2GO$_{\text{Node2vec+SVM}}$	3.99	2.5e-14	1.3e-2
Node2ve+SVM	4.36	<2.2e-16	1.0e-2

Experimental			
Methods	Average Rank	P-value	Adjusted α
STRING2GO$_{\text{Mashup+Sigmoid}}$ (ctrl.)	2.44	N/A	N/A
STRING2GO$_{\text{Node2vec+Sigmoid}}$	2.82	4.0e-2	5.0e-2
Node2ve+SVM	3.76	9.3e-13	2.5e-2
STRING2GO$_{\text{Node2vec+SVM}}$	3.84	3.7e-14	1.7e-2
STRING2GO$_{\text{Mashup+SVM}}$	3.93	8.3e-16	1.3e-2
Mashup+SVM	4.22	<2.2e-16	1.0e-2

Database			
Methods	Average Rank	P-value	Adjusted α
STRING2GO$_{\text{Mashup+Sigmoid}}$ (ctrl.)	2.64	N/A	N/A
STRING2GO$_{\text{Node2vec+Sigmoid}}$	2.82	3.3e-1	5.0e-2
STRING2GO$_{\text{Mashup+SVM}}$	3.49	4.4e-6	2.5e-2
Mashup+SVM	3.70	1.0e-8	1.7e-2
STRING2GO$_{\text{Node2vec+SVM}}$	3.99	2.9e-13	1.3e-2
Node2ve+SVM	4.37	<2.2e-16	1.0e-2

Coexpression			
Methods	Average Rank	P-value	Adjusted α
STRING2GO$_{\text{Mashup+Sigmoid}}$ (ctrl.)	2.68	N/A	N/A
STRING2GO$_{\text{Mashup+SVM}}$	3.01	7.5e-2	5.0e-2
STRING2GO$_{\text{Node2vec+Sigmoid}}$	3.03	5.9e-2	2.5e-2
Mashup+SVM	3.80	1.4e-9	1.7e-2
Node2vec+SVM	4.22	<2.2e-16	1.3e-2
STRING2GO$_{\text{Node2vec+SVM}}$	4.25	<2.2e-16	1.0e-2
